# The actual status of drug prices and adjustment factors for drug price calculation: an analysis of ultra-orphan drug development in Japan

**DOI:** 10.1186/s13023-022-02526-z

**Published:** 2022-11-08

**Authors:** Akihiko Kawakami, Ken Masamune

**Affiliations:** 1 Cooperative Major in Advanced Biomedical Sciences, Joint Graduate School of Tokyo Women’s Medical University and Waseda University, 8-1 Kawadacho, Shinjuku-ku, Tokyo, Japan; 2 Healthcare and Wellness Division, Mitsubishi Research Institute, Inc., 10-3 Nagatacho 2-Chome, Chiyoda-ku, Tokyo, Japan

**Keywords:** Ultra-orphan drugs, Ultra-rare disease, Pricing, Price regulation, Japan

## Abstract

**Background:**

Extremely high prices facilitate drug development for ultra-rare diseases (ultra-orphan drugs). However, various problems arise in terms of healthcare financing and fairness, and the status of ultra-orphan drug pricing remains ambiguous. In this study, we investigated ultra-orphan drug prices in Japan relative to that of other drugs. We examined the relationship between annual expected drug prices and expected sales, and the expected number of patients, for 393 drugs containing new active ingredients for therapeutic use that were listed on the National Health Insurance drug price list in Japan between April 16, 2010 and August 26, 2020. In addition, we compared prices, the drug price calculation method, and price calculation adjustment factors for ultra-orphan and other drugs.

**Results:**

Drug prices tended to increase as the expected number of patients to whom the drug was administered decreased; however, this trend diminished when the expected number of patients was less than 1000. On the other hand, the expected sales tended to decrease as the number of expected patients decreased, and this tendency was reinforced when the expected number of patients was less than 1000. The cost accounting method tended to be used for the price calculation of ultra-orphan drugs, but there were no price differences based on the drug price calculation method. Regarding the price calculation adjustment factors, the premium for usefulness tended to be higher for ultra-orphan drugs. The premium for marketability was higher for non-orphan drugs but did not differ from that for orphan drugs, except for ultra-orphan drugs.

**Conclusions:**

The status of drug prices and expected sales differed beyond a threshold of 1000 expected patients, indicating that recovering the development cost for ultra-orphan drugs is difficult. In addition, the higher premium for usefulness for ultra-orphan drugs reflects the largely unmet need of the associated diseases. Scarcity among orphan drugs is not considered for marketability, highlighting the need for a new framework to promote the development of ultra-orphan drugs.

## Background

In Japan, medicines that meet certain conditions are designated as orphan drugs, and various support measures are provided for the development of drugs for rare diseases and high medical needs[1, 2]. These conditions are as follows: the number of patients with the target disease in Japan is lower than 50,000; there is a high medical need for the drug; and there is potential for drug development. If a drug meets these conditions and is designated as an orphan drug by the Japanese Ministry of Health, Labor and Welfare (MHLW), it will be eligible for various support measures, including subsidies for research and development, tax measures for research and development, priority measures related to regulatory review, extension of market exclusivity, and an additional amount in the price calculation (described below). Research and development grants are also provided prior to orphan drug designation. These various push and pull incentives are set up to promote the development of orphan drugs.

The MHLW calls ultra-rare diseases as those for which the number of patients is particularly low in Japan—less than 1000 (prevalence less than 1 per 100,000); drugs for ultra-rare diseases are called ultra-orphan drugs [3]. Most ultra-rare diseases are hereditary, occur in childhood, and have a poor prognosis. However, as the number of patients with ultra-rare diseases is extremely small, the market for ultra-orphan drugs is limited, and the difficulty in recovering drug development costs is one of the barriers to their development. Therefore, many of the ultra-rare diseases have no cure and largely unmet medical needs [4].

In Japan, under the universal health insurance system, all citizens are entitled to receive insured medical treatment. The National Health Insurance (NHI) prices of new drugs used for insured medical treatment are calculated based on the price standards set by the MHLW [5]. There are three main methods for calculating drug prices: the similar efficacy comparison method, the cost accounting method, and other special cases. The similar efficacy comparison method is applied for new drugs with similar effects, and the price of the new drug is calculated based on that of the most similar comparator drug in terms of efficacy and effectiveness, pharmacological action, composition and chemical structure, dosage form, dosage category, dosage form and dosage form. The similar efficacy comparison method (II) is applied for items that lack novelty, whereas the similar efficacy comparison method (I) is applied for other items. For the latter, an additional premium may be applied from the perspectives of innovation, usefulness, and marketability. When no similar drugs exist, the cost accounting method is applied for pricing by adding the amounts equivalent to the costs of production and sales, general and administrative expenses, operating profit, distribution costs, and local consumption tax. Until March 2018, an operating profit markup was included based on the degree of innovativeness of the new drug for prices calculated using the cost accounting method. In April 2018, the cost accounting method was revised, and the operating profit markup was abolished. Since then, the additional premium has been applied like in the similar efficacy comparison method (I) according to the degree of disclosure of total product costs. The Sakigake Designation Scheme, established in April 2016, mandates an additional premium for drugs developed in Japan ahead of the rest of the world that are expected to show significant efficacy at early clinical trial stages. In addition, the calculated price can be increased or decreased for items that deviate from foreign prices by more than a certain amount (foreign average price adjustment) to prevent significant variations for drugs that are already sold in foreign countries. Therefore, the price of a drug is determined by a variety of factors (hereafter referred to as *price calculation adjustment factors*). Examples of drug price adjustment are shown in Table 1.

**Table 1:** Examples of drug price adjustment

Trade name/Generic name	Tepmetko Tablets 250 mg/Tepotinib Hydrochloride Hydrate	Crysvita Subcutaneous Injection 10 mg 1 mL/Burosumab (Genetical Recombination)
Drug price calculation method	Similar efficacy comparison method (I)	Cost accounting method (new)
Price before adjustment (A)	12,520.90 JPY	155,774.00 JPY
Premium for usefulness-based group (B)	5	45
Premium for marketability-based group (C)	0	10
Premium for Sakigake Designation Scheme (D)	10	0
Addition factor^*1^ (E)	–	1.0
Foreign average price adjustment^*2^ (F)	1	1.26245
Calculation Formula	A × (1 + (B + C + D)/100) × F	A × (1 + (B + C + D)/100 × E) × F
Price	14,399.00 JPY	304,818.00 JPY

*1 Factor used in the case of the cost accounting method (new), depending on the degree of disclosure of total product cost. *2 Calculated as 1 if there is no adjustment

The drug prices of ultra-orphan drugs can be significantly high [6, 7]. As noted earlier, the promotion of research and development of ultra-orphan drugs is being sought through various pull and push incentives, and the significantly higher drug prices of ultra-orphan drugs may be one of the factors encouraging ultra-orphan drug R&D [8]. In 2019, the drug development organizations requested the MHLW to establish an additional charge for ultra-orphan drugs [9]. However, this is expected to have a sizable impact on medical finances under the universal health insurance system. Furthermore, this may evoke resentment as a large amount of tax revenues and insurance premiums will be spent on a small percentage of patients with ultra-rare diseases.

Although the rarity of a disease is inversely correlated with the annual cost of treatment, few studies have focused on the actual prices of ultra-orphan drugs; the differences in drug price calculation adjustment factors between ultra-orphan and other drugs have not been examined in the literature [10, 11].

In this study, we compare the actual status of prices and price calculation adjustment factors for ultra-orphan drugs—that have been approved for production and marketing and listed on the drug price list in Japan—and other drugs.

Analyzing such differences, this study makes a novel contribution by clarifying the status of ultra-orphan drug prices in Japan and has implications for the future development of ultra-orphan drugs and the optimization of healthcare finances.

The remainder of this paper is structured as follows. Sections 2 and 3 present the methods and the key results, respectively. Section 4 presents a discussion, and Sect. 5 concludes the paper.

## Methods

### Research context

Drugs containing new active ingredients for therapeutic use—excluding diagnostic drugs and anesthetics—that were listed between April 16, 2010 and August 26, 2020 were extracted from the data on new NHI drug price listings published by the General Assembly of the Central Social Insurance Medical Council of the MHLW, Japan [12].

We identified whether the sampled drugs were orphan drugs by referring to the list published by the National Institute of Biomedical Innovation, Health and Nutrition [13]. For orphan drugs designated prior to September 2019, ultra-orphan drugs were identified by referring to the Survey on the Development Trend of Ultra-Orphan Drugs published by the Japan Agency for Medical Research and Development [14]. For orphan drugs designated after October 2019, we examined whether the number of patients in Japan using the drug in question was less than 1000, based on the minutes of the deliberations at the First and Second Committees of the Pharmaceutical Affairs and Food Sanitation Council of the MHLW [15, 16]. The sample drugs that were designated as orphan drugs and have fewer than 1000 patients in Japan were classified as *ultra-orphan drugs*, drugs with more than 1000 patients were classified as *orphan drugs*, and drugs not designated as orphan drugs were classified as *non-orphan drugs*.

For the entire sample, we collected information on the drug price calculation method, the additional premium, the operating profit markup, the foreign average price adjustment rate, the expected number of patients administered during the peak period, and the expected sales during the peak period. The ratio of the expected sales to the expected number of patients administered was used as the “annual expected drug price.”

### The relationships of the annual expected drug price and the expected sales with the expected number of patients

To examine the relationships between the annual expected drug price and the expected sales of drugs containing the new active ingredient, and the expected number of patients who received the drug containing the new active ingredient, we conducted the Kruskal–Wallis test of the annual expected drug prices for seven groups:0–9 patients, 10–99 patients, 100–999 patients, 1000–9999 patients, 10,000–99,999 patients, 100,000–999,999 patients, and more than 1,000,000 patients; multiple comparisons were performed using the Steel–Dwass method.

### Comparisons of the price calculation status of ultra-orphan and other drugs

The annual expected drug prices, expected sales, drug price calculation methods, and price calculation adjustment factors of ultra-orphan, orphan, and non-orphan drugs were compared between groups using the Kruskal–Wallis test. Multiple comparisons were made using the Steel–Dwass method.

The comparisons involved the following main pricing methods: a) the similar efficacy comparison method (I); b) the cost accounting method after the April 2018 revision (cost accounting method (new)), wherein an additional premium is applied; and c) the cost accounting method before the April 2018 revision (cost accounting method (old)), wherein an operating profit markup is applied. Additional premiums were categorized into the “premium for usefulness-based group”, which included premiums for innovation, usefulness (I), and usefulness (II), and the “premium for marketability-based group”, which included premiums for marketability (I), marketability (II), and pediatric use and pertained to drugs with low marketability such as rare drugs and drugs for pediatric use. The comparison focused on items placed on the NHI drug price list after April 2016, when the Sakigake system was established.

Statistical software (JMP Pro 15.0.0, SAS) was used for the analysis of the basic statistics at the 10% level of significance.

## Results

### Research context

**Fig. 1:**
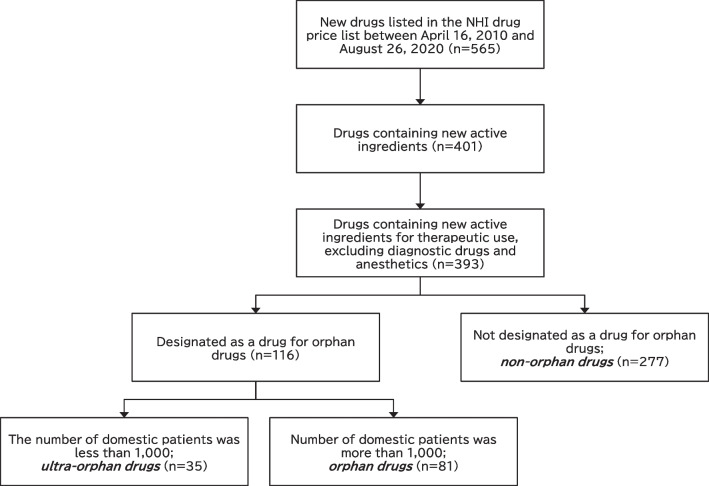
Classification of research items

In Japan, there were 565 new drugs listed on the NHI drug price list between April 16, 2010 and August 26, 2020. Of these, 393 drugs were new active ingredients for therapeutic use, excluding diagnostic drugs and anesthetics. Of the 393 drugs, 35 drugs were ultra-orphan drugs, 81 drugs were orphan drugs, and 277 drugs were non-orphan drugs (Fig. 1).

### Comparison of the annual expected drug price and the expected sales with the expected number of patients

#### Annual expected drug price

**Fig. 2:**
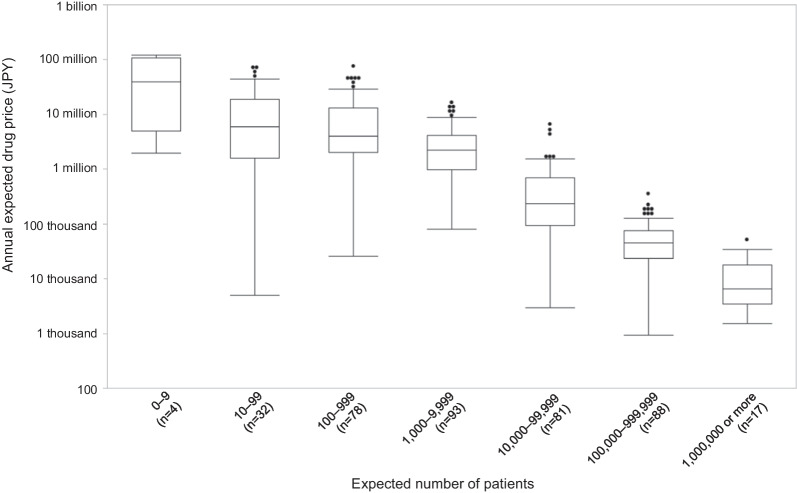
The relationship between annual expected drug prices and the expected number of patients in Japan

The 393 drugs containing the new active ingredient for therapeutic use were divided into the seven patient groups with 4, 32, 78, 93, 81, 88, and 17 drugs, respectively. Figure 2 shows a box and whisker plot of the annual expected drug prices by the expected number of patients.

There are significant differences in annual expected drug prices between the seven groups (*p* < 0.0001). Table 2 shows the presence (or absence) of significant differences when multiple comparisons are made. There is no significant difference between the three groups with less than 1000 expected patients that include ultra-orphan drugs. When comparing each group with fewer than 1000 people to each group with more than 1000 people, there is no significant difference between the 0–9 and 1000–9999 groups, and between the 10–99 and 1000–9999 groups, unlike between the other groups. On the other hand, there is a significant difference between all four groups with more than 1000 expected patients.

**Table 2:** The differences in annual expected drug prices by the expected number of patients

Expected number of patients	Conjunctive letter of the annual expected drug price				
0–9	A	B			
10–99	A	B			
100–999	A				
1000–9999		B			
10,000–99,999			C		
100,000–999,999				D	
1,000,000 or more					E

There is a significant difference between groups that are not connected by the same letter. For example, the three groups of 0–9, 10–99, and 100–999 are connected by A because there is no significant difference between them. On the other hand, the group of 1000–9999 is connected by B instead of A because there is no significant difference between the three groups of 0–9, 10–99 and 1000–9999 but there is a significant difference between the 100–999 group and the 1000–9999 group

#### Expected sales

**Fig. 3:**
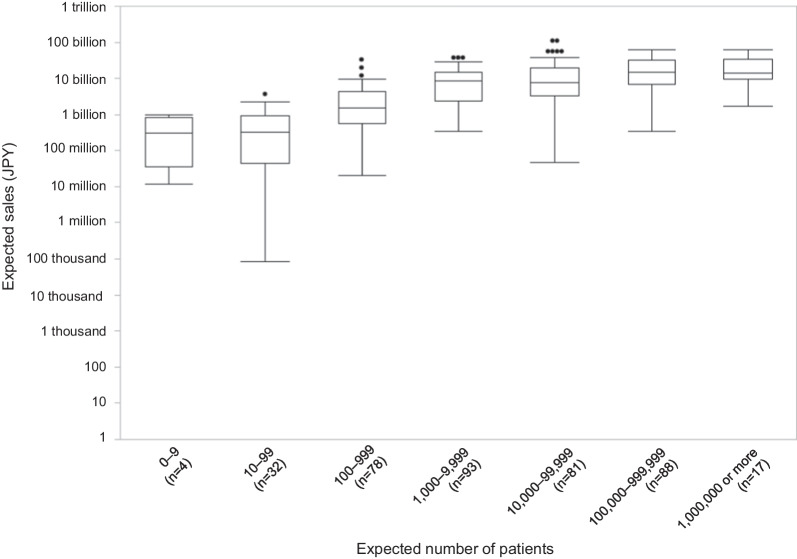
The relationship between the expected sales and the expected number of patients in Japan

Figure 3 shows a box and whisker plot of the expected sales of 393 drugs containing new active ingredients for therapeutic use by the number of expected patients.

There is a significant difference of the expected sales between the seven groups. Table 3 shows the presence (or absence) of significant differences when multiple comparisons are made. Of the three groups with less than 1000 expected patients that include ultra-orphan drugs, there is a significant difference between the 10–99 and 100–999 groups, but none between the other groups. When comparing each group with fewer than 1000 people to each group with more than 1000 people, significant differences are found. On the other hand, for the four groups with more than 1000 expected patients, there is a significant difference between the 1000–9999 and 100,000–999,999 groups, between the 1000–9999 and 1,000,000 or more groups, and between the 10,000–99,999 and 100,000–999,999 groups, but none between the other groups.

**Table 3:** The differences in the expected sales by the expected number of patients

Expected number of patients	Conjunctive letter of the expected sales				
0–9	A				
10–99	A				
100–999		B			
1000–9999			C		
10,000–99,999			C	D	
100,000–999,999					E
1,000,000 or more				D	E

There is a significant difference between groups that are not connected by the same letter. For example, the 0–9 group is not significantly different from the 10–99. On the other hand, there is a significant difference between the 100–999 group and any other groups. Therefore, the 0–9 group is connected to the 10–99 group by A, but the 100–999 group is not connected to any other groups by B

### Comparison of price calculation status between ultra-orphan and other drugs

#### Annual expected drug price

**Fig. 4:**
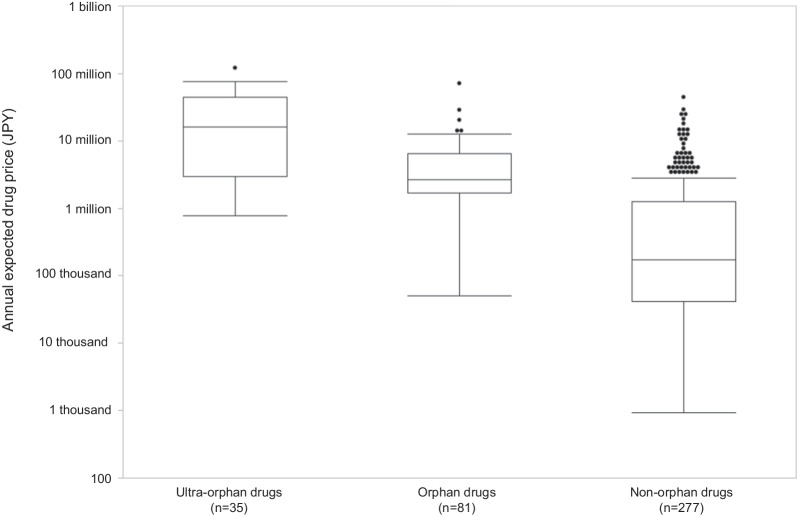
Comparison of annual expected drug prices for ultra-orphan and other drugs in Japan

Box and whisker plots of the annual expected drug prices for ultra-orphan, orphan, and non-orphan drugs are shown in Fig. 4. The annual expected drug prices of ultra-orphan drugs are significantly higher than that of orphan and non-orphan drugs (*p* < 0.0001). The annual expected drug prices of orphan drugs are also significantly higher than that of non-orphan drugs (*p* < 0.0001).

#### Expected sales

**Fig. 5:**
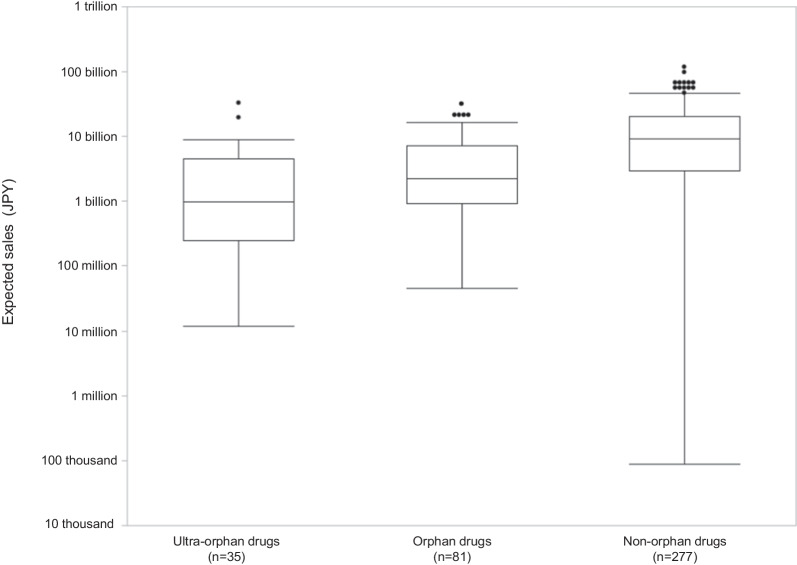
Comparison of the expected sales of ultra-orphan and other drugs in Japan

Box and whisker plots of the expected sales for ultra-orphan, orphan, and non-orphan drugs are shown in Fig. 5. The expected sales of ultra-orphan drugs are significantly smaller than that of orphan and non-orphan drugs (*p* = 0.0152 and *p* < 0.0001, respectively). The expected sales of orphan drugs are also significantly smaller than that of non-orphan drugs (*p* < 0.0001).

#### Drug price calculation method

**Fig. 6:**
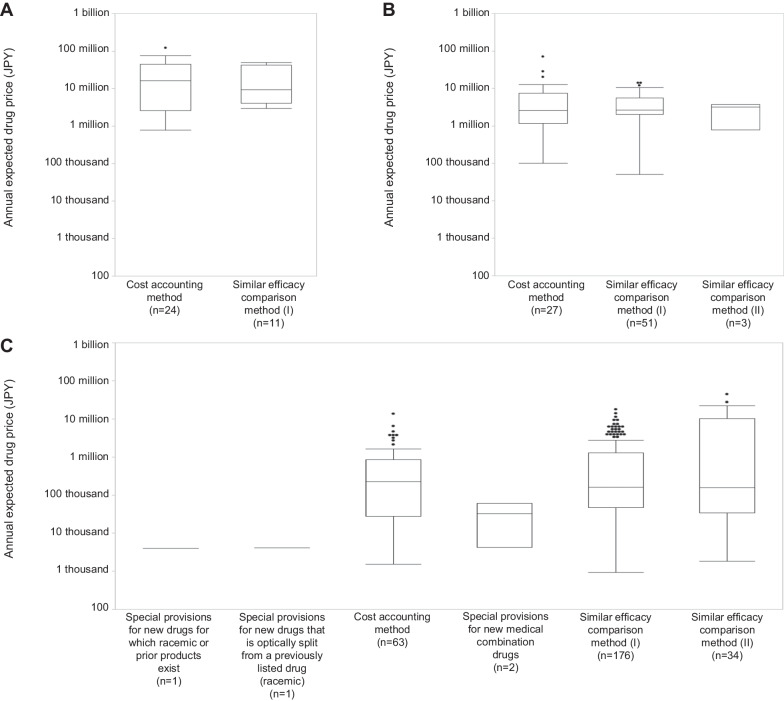
Comparison of the annual expected drug prices by the drug price calculation method. **A** Ultra-orphan drugs. **B** Orphan drugs. **C** Non-orphan drugs

The drug price calculation methods applied for ultra-orphan, orphan, and non-orphan drugs are shown in Table 4. The only methods applied for ultra-orphan drugs are the similar efficacy comparison method (I) and the cost accounting method. The percentage of application of the similar efficacy comparison method (I) is higher than that of the cost accounting method for orphan and non-orphan drugs, whereas the percentage of application of the cost accounting method is higher than that of the similar efficacy comparison method (I) for ultra-orphan drugs. There is no difference in the annual expected drug prices for ultra-orphan, orphan, and non-orphan drugs due to differences in the NHI price calculation method (Fig. 6).

**Table 4:** The various drug price calculation methods applied

Drug price calculation method	Ultra-orphan drugs		Orphan drugs		Non-orphan drugs	
All	35	(100%)	81	(100%)	277	(100%)
Similar efficacy comparison method (I)	11	(31.4%)	51	(63.0%)	176	(63.5%)
Similar efficacy comparison method (II)	0	(0%)	3	(3.7%)	34	(12.3%)
Cost accounting method	24	(68.6%)	27	(33.3%)	63	(22.7%)
Special provisions for new medical combination drugs	0	(0%)	0	(0%)	2	(0.7%)
Special provisions for new drugs for which racemic or prior products exist	0	(0%)	0	(0%)	1	(0.4%)
Special provisions for new drugs that are optically split from a previously-listed drug (racemic)	0	(0%)	0	(0%)	1	(0.4%)

#### Price calculation adjustment factors

We compared the price calculation adjustment factors for the similar efficacy comparison method (I), the cost accounting method (new), and the cost accounting method (old) between the ultra-orphan, orphan, and non-orphan drug groups.

#### Similar efficacy comparison method (I)

Table 5 shows the application status of the price calculation adjustment factors for the 238 drugs using the similar efficacy comparison method (I). A comparison of the usefulness-based group premiums shows a significant difference between the ultra-orphan, orphan, and non-orphan drug groups (*p* = 0.0193). In multiple comparisons, there is a significant difference between the ultra-orphan and non-orphan drug groups (*p* = 0.0201), but none between the other groups (ultra-orphan drug–orphan drug, *p* = 0.1382; orphan drug–non-orphan drug, *p* = 0.4717). For the marketability-based group premiums, there is a significant difference between the three groups (*p* = 0.0002). In multiple comparisons, there is a significant difference between the orphan drug and non-orphan drug groups (*p* = 0.0002), but none between the other groups (ultra-orphan drug–orphan drug, *p* = 0.9703; ultra-orphan drug–non-orphan drug, *p* = 0.1061). Furthermore, for the premiums for the Sakigake Designation Scheme, there is a significant difference between the orphan drug and non-orphan drug groups (*p* = 0.0561). In multiple comparisons, there is a significant difference between the orphan drug and non-orphan drug groups (*p* = 0.0670), but none between the other groups (ultra-orphan drug–orphan drug, *p* = 0.5940; ultra-orphan drug–non-orphan drug, *p* = 0.9510). There is no significant difference between the three groups in terms of the foreign average price adjustment rate (*p* = 0.7598).

**Table 5:** Status of application of drug price calculation adjustment factors in the similar efficacy comparison method (I)

	Ultra-orphan drugs	Orphan drugs	Non-orphan drugs
Premium for usefulness-based group	n = 11	n = 51	n = 176
100%	0	0	1
75%	0	0	0
50%	1	0	0
45%	2	0	0
40%	1	1	2
35%	0	1	1
20%	0	1	1
15%	0	1	1
10%	0	4	12
5%	2	8	24
0%	5	35	134
Premium for marketability-based group	n = 11	n = 51	n = 176
10%	2	10	2
5%	0	1	6
0%	9	40	168
Premium for Sakigake Designation Scheme	n = 9	n = 28	n = 78
10%	0	3	1
0%	9	25	77
Foreign average price adjustment	n = 11	n = 51	n = 176
100% raise	0	3	7
90–99% raise	0	0	1
80–89% raise	0	0	0
70–79% raise	0	0	1
60–69% raise	0	0	2
50–59% raise	1	0	1
40–49% raise	0	1	0
30–39% raise	0	1	2
20–29% raise	0	0	2
10–19% raise	0	1	4
1–9% raise	1	5	3
Not applicable	9	36	147
1–9% lowering	0	1	3
10–19% lowering	0	1	1
20–29% lowering	0	2	1
30–39% lowering	0	0	0
40–49% lowering	0	0	1
50–59% lowering	0	0	0
60–69% lowering	0	0	0
70% and more lowering	0	0	0

#### Cost accounting method (new)

Table 6 shows the application status of the price calculation adjustment factors for the 27 drugs using the cost accounting method (new).

**Table 6:** Status of application of drug price calculation adjustment factors in the cost accounting method (new)

	Ultra-orphan drugs	Orphan drugs	Non-orphan drugs
	n = 6	n = 7	n = 14
*Premium for usefulness-based group*			
100%	0	0	0
75%	0	2	0
50%	0	0	0
45%	1	0	1
40%	0	0	1
35%	0	1	1
20%	0	0	1
15%	0	0	0
10%	1	2	0
5%	3	2	1
0%	1	0	9
*Premium for marketability-based group*			
10%	6	7	0
5%	0	0	2
0%	0	0	12
*Premium for Sakigake designation scheme*			
10%	1	0	1
0%	5	7	13
*Foreign average price adjustment*			
100% raise	0	0	0
90–99% raise	0	0	0
80–89% raise	0	0	0
70–79% raise	0	0	0
60–69% raise	0	0	0
50–59% raise	0	0	0
40–49% raise	0	0	0
30–39% raise	0	0	0
20–29% raise	1	0	0
10–19% raise	0	0	0
1–9% raise	0	0	1
Not applicable	5	7	13
1–9% lowering	0	0	0
10–19% lowering	0	0	0
20–29% lowering	0	0	0
30–39% lowering	0	0	0
40–49% lowering	0	0	0
50–59% lowering	0	0	0
60–69% lowering	0	0	0
70% and more lowering	0	0	0

A comparison of the usefulness-based group premiums shows a significant difference between the ultra-orphan, orphan, and non-orphan drug groups (*p* = 0.0456). In multiple comparisons, there is a significant difference between the orphan drug and non-orphan drug groups (*p* = 0.0618), but none between the other groups (ultra-orphan drug–orphan drug, *p* = 0.3376; ultra-orphan drug–non-orphan drug, *p* = 0.4298). For the marketability-based group premiums, there is a significant difference between the three groups (*p* < 0.001). In multiple comparisons, there is a significant difference between the ultra-orphan and non-orphan and between the orphan and non-orphan drug groups (*p* = 0.0002 and *p* = 0.0001, respectively), but none between the ultra-orphan and orphan drug groups (*p* = 1.000). Furthermore, there is no significant difference between the three groups in terms of the premiums for the Sakigake Designation Scheme and the foreign average price adjustment rate (*p* = 0.5318 and *p* = 0.5055, respectively).

#### Cost accounting method (old)

Table 7 shows the application of the price calculation adjustment factors for the 87 drugs using the cost accounting method (old). There is no significant difference between the ultra-orphan, orphan, and non-orphan drug groups in terms of the operating profit markup (*p* = 0.3812). For the foreign average price adjustment rate, there is a significant difference between the ultra-orphan, orphan, and non-orphan drug groups (*p* = 0.0527). In multiple comparisons, there is a significant difference between the ultra-orphan drug and orphan drug groups (*p* = 0.0439), but none between the other groups (ultra-orphan drug–non-orphan drug, *p* = 0.5575; orphan drug–non-orphan drug, *p* = 0.1818). Of the 18 ultra-orphan drugs, three were lowered and none were raised. Of the 20 orphan drugs, three were raised and none were lowered.

**Table 7:** Status of application of drug price calculation adjustment factors in the cost accounting method (old)

	Ultra-orphan drugs	Orphan drugs	Non-orphan drugs
	n = 18	n = 20	n = 49
*Operating profit markup*			
60%	0	1	0
50%	0	0	1
40%	0	1	1
35%	0	1	0
30%	2	0	1
25%	0	1	0
20%	2	3	5
10%	1	2	9
0%	13	11	31
−5%	0	0	1
*Foreign average price adjustment*			
100% raise	0	0	0
90–99% raise	0	0	1
80–89% raise	0	0	0
70–79% raise	0	0	0
60–69% raise	0	1	0
50–59% raise	0	0	1
40–49% raise	0	0	0
30–39% raise	0	1	0
20–29% raise	0	0	0
10–19% raise	0	1	0
1–9% raise	0	0	1
Not applicable	15	17	41
1–9% lowering	1	0	0
10–19% lowering	1	0	1
20–29% lowering	0	0	0
30–39% lowering	0	0	3
40–49% lowering	0	0	0
50–59% lowering	1	0	0
60–69% lowering	0	0	1
70% and more lowering	0	0	0

## Discussion

Similar to previous studies in the European context, the present findings showed that the annual price of a new drug in Japan increased with a decrease in the expected number of patients [10, 11]. Furthermore, this study found that the trend became less pronounced as the expected number of patients decreased; the annual expected drug price did not change with a change in the expected number of patients when the latter, which included the ultra-orphan drug group, was less than 1000. The expected sales tended to be lower as the expected number of patients decreased, especially when the number was less than 1000. However, this tendency was weakened when the expected number of patients exceeded 1000. Based on these results, we propose that 1000 patients be set as the threshold for ultra-orphan drug pricing.

The annual expected drug prices and the expected sales of ultra-orphan drugs were significantly higher and lower, respectively, than those of orphan and non-orphan drugs. Although the annual expected drug prices of ultra-orphan drugs are set high, they do not fully compensate for the low number of patients treated, and the market for ultra-orphan drugs is smaller than that for orphan and non-orphan drugs, suggesting that it is difficult to recover the drug development cost.

In terms of the drug price calculation methods applied, the results showed that the cost accounting method tended to be applied more in the case of ultra-orphan drugs compared to orphan and non-orphan drugs. However, there was no difference in the annual expected drug prices across price calculation methods for ultra-orphan, orphan, and non-orphan drugs. The difference in the ratio of the drug price calculation methods applied was thus considered ineffective in raising the annual ultra-orphan drug prices.

The usefulness-based group premium in the similar efficacy comparison method (I) was significantly higher for ultra-orphan drugs than for non-orphan drugs, whereas in the cost accounting method (new), orphan drugs were significantly higher than non-orphan drugs, and ultra-orphan drugs also tended to be higher, although not significantly so. The cost accounting method (new) is still relatively nascent and includes only a few drugs, presenting scope for further research. Based on the trend, ultra-orphan drugs tend to have a higher usefulness-based group premium, which may be reflective of the fact that many of them are for diseases with largely unmet needs.

There was no significant difference between ultra-orphan and other drugs in terms of the marketability-based group premium in the similar efficacy comparison method (I). This is because, in cases where the comparator drug is subject to the marketability (I) premium, its price, which forms the basis for the new drug’s price, already reflects such a premium. In the cost accounting method (new), the premium for ultra-orphan drugs was significantly higher compared to that for non-orphan drugs. The marketability premium (I) of 10 points was applied to all ultra-orphan and orphan drugs, and marketability was considered for orphan drugs without similar drugs, according to the application criteria. However, there were no drugs for which the upper limit of 20 points was applied, suggesting that the difference in rarity between ultra-orphan and orphan drugs was not considered.

In the cost accounting method (old), there is no difference between ultra-orphan and other drugs in terms of the operating profit markup, implying that the drug prices do not sufficiently reflect the usefulness and marketability of ultra-orphan drugs.

Regarding the foreign average price adjustment, if the NHI-based price calculated for Japan is lower than that of the other countries, this may divert drug development overseas; however, in the similar efficacy comparison method (I) and the cost accounting method (new), there were no differences in the foreign average price adjustment rates of ultra-orphan, orphan, and non-orphan drugs. On the other hand, in the cost accounting method (old), there was a significant difference between ultra-orphan drug and orphan drug, but the reason for this difference needs to be confirmed for individual items. It should be noted that this study only compared the adjustment rates. The relationship between ultra-orphan drug prices and drug lags can be further clarified by comparing ultra-orphan drug prices in Japan with those in other countries, and comparing the timings of marketing approval, drug price listing, and insurance reimbursement.

The premium for the Sakigake Designation Scheme established in April 2016 has been applied to only 7 of the 393 drugs surveyed, making it difficult to assess its effect on ultra-orphan drug development at present. However, of the seven drugs covered by the premium, one is an ultra-orphan drug, and four are orphan drugs, which may provide incentives for the development of drugs for rare diseases, including ultra-orphan drugs, in Japan.

The cost accounting method (new) is only applied to drugs listed on the NHI drug price list as new drugs after the April 2018 revision, and is not retroactively applied to drugs whose prices were calculated using the cost accounting method (old) before that date. Therefore, the number of drugs calculated under the cost accounting method (new) is limited, and the analysis of the results is also limited. If the cost accounting method (new) were applied retroactively to items for which NHI drug prices were calculated using the old method, the difference in scores for the premium for usefulness-based group would be more pronounced, as shown in Similar efficacy comparison method (I). On the other hand, for the premium for marketability-based group, it is not applied to distinguish between ultra-orphan drugs and orphan drugs, so the results would have been similar to the present results. These speculations can be verified when the number of items calculated under the cost accounting method (new) increases and the number of items becomes sufficient for analysis.

Most negotiations and discussions between pharmaceutical companies and NHI pricing organizations about the calculation of NHI prices for new drugs are not publicly disclosed. The scope of this study was limited by its use of publicly available information on NHI pricing. It should also be noted that the annual expected drug prices, expected sales and expected number of patients used in this study are calculated values, not actual values. In addition, this study did not examine the differences in modalities, novelty of the mechanisms of action, existence of existing treatments, severity of disease, clinical trial results, and study designs used in the applications for approval. Specifically, for the development of ultra-orphan drugs, the number of cases and the study design—such as the presence of a control group—are limited. Therefore, it is necessary to examine how these factors affect the NHI prices and price calculation adjustment factors to promote the development of ultra-orphan drugs in Japan in the future.

## Conclusions

In this study, we analyzed the actual status of NHI prices and drug price adjustment factors for ultra-orphan drugs in Japan. A threshold of 1000 patients emerged as the standard for ultra-orphan drugs from the perspective of drug pricing. Although the annual expected drug prices of ultra-orphan drugs are higher than that of orphan drugs, the expected sales are smaller, indicating that it is more difficult to recover development costs. In addition, the premiums for innovation and usefulness are higher for ultra-orphan drugs, which reflects the fact that many of the drugs are for diseases with largely unmet needs. However, the premium for marketability does not consider rarity or the differences between ultra-orphan and orphan drugs. Therefore, a new framework is required to promote the development of ultra-orphan drugs.

Additionally, this study reveals that the use of the revised cost accounting method allows the usefulness and marketability of ultra-orphan drugs to be better reflected in NHI prices and that there is no difference in the average foreign price adjustment rate between ultra-orphan and other drugs. These insights can contribute to the introduction and promotion of ultra-orphan drugs that are developed overseas in Japan.

## Data Availability

The datasets used and/or analyzed in the current study are available from the corresponding author on reasonable request.

## Abbreviations

MHLW: Ministry of Health, Labor and Welfare
NHI: National Health Insurance
ANOVA: Analysis of variance
